# Acoustic-based Stenosis Detection for Dialysis Patients using Explainable Machine Learning

**DOI:** 10.21203/rs.3.rs-9781692/v1

**Published:** 2026-05-22

**Authors:** Mohsen Annabestani, George Zhou, Herrick Wun, Bobak Mosadegh

**Affiliations:** 1. Dalio Institute of Cardiovascular Imaging, Department of Radiology, Weill Cornell Medicine, NY, USA; 2. Kaiser Permanente Southern California, Los Angeles Medical Center, Los Angeles, CA, USA; 3. Department of Vascular Surgery, NewYork-Presbyterian Hospital, New York, NY, USA, USA

## Abstract

Ensuring the long-term patency of arteriovenous fistulas (AVFs) is essential for patients undergoing hemodialysis, yet existing monitoring methods often lack the accessibility and interpretability required for routine point-of-care use. In this study, we performed a comparative evaluation of classical machine learning (ML) models versus a state-of-the-art Vision Transformer (ViT) deep learning (DL) architecture for automated detection of AVF stenosis using non-invasive acoustic recordings. Our approach employed a comprehensive pipeline combining expert-designed acoustic features—such as Mel-frequency cepstral coefficients (MFCCs) and peak amplitude—with anatomical metadata to train a range of classical classifiers. These models were compared against a ViT trained on Mel-spectrogram representations of the same recordings. The results show that classical ML models, whether applied to individual anatomical sites or using universal models informed by anatomical context, consistently outperformed the ViT deep model. At the patient level, both approaches achieved comparable performance, with an F1 score of 0.91. Importantly, integrating Explainable AI (XAI) through SHAP analysis demonstrated that classical models base their predictions on physiologically meaningful features—such as elevated signal energy and spectral shifts—that reflect the hemodynamic turbulence associated with stenosis. By combining high precision with interpretability, classical ML offers a clinically reliable framework for early-stage AVF monitoring, with the potential to enhance long-term vascular access outcomes.

## 1. Introduction

Reliable vascular access is essential for maintenance hemodialysis, with arteriovenous fistulas (AVFs) considered the gold standard due to superior long-term patency and lower complication rates compared with grafts or catheters. The National Kidney Foundation consistently emphasizes vascular access as a critical determinant of dialysis effectiveness and patient outcomes [1–3]. However, maintaining functional AVFs remains challenging, with dysfunction leading to repeated interventions, increased morbidity, and substantial healthcare costs. Early patency loss has been reported at 19.7%, rising to over 33% during long-term follow-up [4, 5], while registry data indicate that more than half of AVFs lose primary unassisted patency within the first year [6]. The main causes of failure are stenosis and thrombosis [6–11]; focal vessel narrowing alters hemodynamics and frequently precedes thrombotic occlusion. Incidence rates range from 4.6–10.8% for stenosis and 2.3–7.7% for thrombosis [12], with most thrombosed accesses harboring underlying stenotic lesions[13]. These complications are a major source of hospitalization among dialysis patients[14], and recent studies highlight persistent gaps in prevention and monitoring strategies [15–17]. Early detection of stenosis is crucial to prolonging AVF lifespan and reduce clinical and economic burden[18–21]. Current KDOQI guidelines emphasize routine monitoring primarily via physical examination, assessing changes in bruit, thrill, bleeding duration, and limb swelling [22–24]. The 2019 update removed recommendations for flow and pressure surveillance, reflecting limitations in existing methods and highlighting the need for reliable, device-based monitoring solutions. Emerging research increasingly focuses on objective, technology-driven approaches, including wearable sensors and continuous monitoring systems, which may improve early detection and intervention [25–27].

Auscultation provides a simple, non-invasive method to assess vascular flow, where laminar flow in healthy AVFs generates stable acoustic patterns, and stenosis introduces turbulence with higher frequencies and irregular signals. These acoustic signatures can indicate early dysfunction. However, auscultation is operator-dependent and prone to variability, limiting reliability[28, 29]. To address these limitations, recent work has applied AI and signal processing to automate bruit analysis, improving consistency and potentially enabling earlier identification of high-risk access sites[30, 31]. In particular, our previous work demonstrated that deep learning models [32], specifically Vision Transformers (ViT) [33], can effectively detect AVF stenosis by analyzing spectrogram representations of blood flow sounds. While this framework achieved strong performance across multiple anatomical locations using duplex ultrasound as ground truth, these cutting-edge architectures function as “black boxes,” lacking the transparency required to understand how predictions relate to underlying physiological mechanisms. This lack of interpretability presents a significant challenge for clinical adoption, as establishing trust and accountability is essential in medical settings. In the present study, we directly build upon this work using the exact same dataset and evaluation framework to enable a rigorous comparison. However, to overcome previous limitations, we investigate classical machine learning models based on expert-engineered acoustic features with physical and biological relevance. By prioritizing explainability, we aim to bridge the gap between automated detection and clinical reality, ensuring that model decisions are grounded in hemodynamic phenomena that clinicians can trust.

While deep learning models can capture complex patterns, their performance often degrades on smaller, structured datasets and when fine-grained predictions at individual sites are required rather than aggregated outcomes. In several studies, high patient-level accuracy (~90%) did not translate to high accuracy at individual access sites, suggesting limitations in generalizability for certain deep networks[34–36]. In contrast, classical machine learning models, especially when informed by identifiable, handcrafted features, have demonstrated competitive or superior performance for structured and limited data contexts. Benchmarks across numerous tabular datasets show that traditional methods such as gradient boosting and random forests frequently match or outperform deep learning models in these settings, particularly when dataset size is modest and feature engineering is feasible[37–39]. This advantage arises because classical models are less prone to overfitting and can leverage domain-specific features more effectively than deep networks on small or mid-sized datasets [37–39]. There is growing recognition that explainable artificial intelligence (XAI) is critical for safe and reliable deployment of AI in healthcare[40–43]. Methods such as SHAP[44] and LIME [36] further support interpretation by revealing how feature inputs influence predictions. In our work, we show that classical approaches not only achieve performance comparable to deep learning but also provide clearer links between acoustic features and hemodynamic phenomena such as turbulence and spectral energy redistribution, offering both accurate site-level predictions and clinically meaningful insights that support transparent AVF monitoring.

## 2. Material and Method

### 2.1 Data

**Figure 1:**
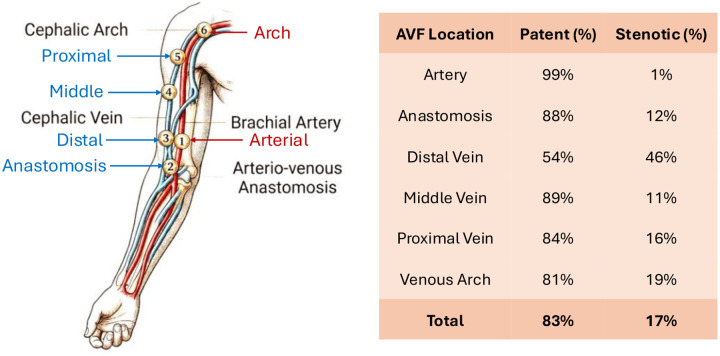
Six anatomical recording sites along the arteriovenous fistula, ordered from distal to proximal: artery, anastomosis, distal vein, middle vein, proximal vein, and venous arch. (Left)The illustration shows a brachiocephalic fistula, while brachiobasilic, radiocephalic, and radiobasilic configurations are also included in the study. (Right) Distribution of patent and stenotic lesions across anatomical AVF locations. *A brachiocephalic arteriovenous fistula (AVF) is presented here as a representative example. However, AVFs can also be created using other vascular configurations, such as connecting the radial artery to the basilic vein.

This study utilizes the exact same dataset as reported by George Zhou et al.[32], enabling a direct and controlled comparison between classical machine learning and deep learning approaches. The dataset consists of blood flow sound recordings collected from patients with AVFs, with corresponding labels (patent vs. stenotic) established using duplex ultrasound as the clinical ground truth. A total of 2,565 AVF sound recordings were obtained from 433 patients, including 2113 patent and 452 stenotic samples. The patient cohort had a mean age at AVF creation of 66.1 ± 13.9 years, with a median age of 67 years (range: 18–91 years). The population consisted of 62.4% male (n = 1601) and 37.6% female (n = 964) patients, reflecting a representative hemodialysis population. Other clinical and demographic characteristics—including body mass index, AVF type, comorbidities, and clinical history—are identical to those reported in [32] and have been provided in the **Table SI.1.1** of Supplementary Information file. Recordings were acquired from multiple anatomical locations along the AVF (Figure 1-**Left**), defined according to standard segmentation of the arm. The proportion of patent and stenotic lesions differs notably across anatomical locations, leading to varying degrees of class imbalance (see Figure 1-**Right**).

### 2.2 Method

**Figure 2:**
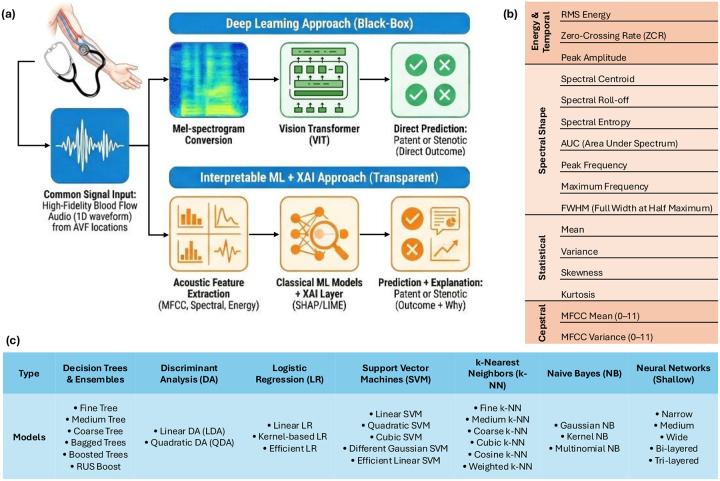
(a) Overview of the two parallel approaches for classifying patent versus stenotic AVFs from high-fidelity blood flow audio signals. The upper path uses a deep learning (black-box) pipeline with mel-spectrogram conversion and Vision Transformer (ViT) for direct prediction[32]. The lower path employs an interpretable pipeline to extract acoustic features (b), modeling through classical ML models (c), and XAI (SHAP/LIME) for transparent prediction and explanation.

As depicted in Figure 2-a, we developed two parallel approaches for this classification problem. The first approach employs a Vision Transformer (ViT) model, which was reported as the best-performing deep learning model in [32], as we use the same dataset in this study. The second approach employs classical machine learning methods combined with a XAI framework to assess the importance of input features. The primary objective of this study is to demonstrate that classical machine learning methods can achieve performance comparable to state-of-the-art deep learning models while offering greater transparency, enabling model interpretability, which is essential for clinical AI applications. Both approaches use the same input signal—high-fidelity blood flow audio from AVF locations. In the deep learning approach, this signal is transformed into a 374×128 two dimensional mel-spectrogram as an image representation, whereas in the classical machine learning approach, it is converted into a set of acoustic features with mechanical, physical, and biological relevance, facilitating interpretation of the model’s decisions.

#### 2.2.1 Acoustic Feature Extraction and Representation

To capture fine-grained acoustic structure and variability associated with turbulence and flow disturbances in vascular signals, an extended set of acoustic features was extracted (Figure 2-b). These include energy and temporal descriptors such as RMS energy, zero-crossing rate (ZCR), and peak amplitude; spectral shape features including spectral centroid, roll-off, entropy, area under the average frequency spectrum (AUC), peak frequency, maximum frequency, and full width at half maximum (FWHM); statistical moments (mean, variance, skewness, and kurtosis); and cepstral features represented by the mean and variance of MFCC coefficients (0–11). Together, these features provide a comprehensive representation of both the temporal dynamics and frequency-domain characteristics of the signal.

This feature set was designed to support both conventional signal processing and similarity-based analysis methods. Spectral features enable identification of frequency distribution changes and broadband turbulence patterns through Fourier or wavelet analysis, while energy and temporal features facilitate envelope tracking and time-series alignment using techniques such as Dynamic Time Warping (DTW). Cepstral features capture global spectral envelop variations, complementing frequency-based analysis, and statistical moments provide additional descriptors of signal irregularity and asymmetry. Overall, this framework encodes both global turbulence-related properties and localized spectral-temporal variations, providing a robust basis for distinguishing between patent and stenotic vascular acoustic signals[45, 46].

#### 2.2.2 Multi-Model Benchmarking of Classical Learning Algorithms

To fully evaluate the potential of classical machine learning, we systematically tested 33 non-deep learning ML models spanning a broad range of algorithmic families (Figure 2-c). These included decision trees and ensemble methods (fine/medium/coarse trees, bagged trees, boosted trees, and RUSBoost), linear and quadratic discriminant analysis, logistic regression (linear and kernel-based), support vector machines (linear, quadratic, cubic, and Gaussian kernels), k-nearest neighbors with multiple distance metrics, Naive Bayes variants, and shallow as well as multi-layer neural networks. This diverse model set is considered comprehensive because it captures fundamentally different learning paradigms, including linear vs. nonlinear decision boundaries, parametric vs. non-parametric approaches, probabilistic vs. distance-based methods, and single-model vs. ensemble strategies. As a result, it enables a robust and unbiased assessment of classical machine learning performance, ensuring that the comparison against deep learning models is not limited by model selection but instead reflects the full capability of traditional approaches.

#### 2.2.3 Local and Global Explainability

To interpret the decision-making mechanisms of the classical machine learning models and to link their predictions to physically and clinically meaningful acoustic phenomena, both global and local explainability analyses were conducted using Shapley values (SHAP) [44] and Local Interpretable Model-agnostic Explanations (LIME) [36]. These analyses were performed for both the location-specific Distal Vein model and the Universal model, which incorporates anatomical metadata, representing the best-performing location-based and universal models in this study.

#### 2.2.3 Classification Scenarios

In our analysis, model performance is evaluated across multiple clinically relevant scenarios to provide a comprehensive assessment of both classical machine learning and deep learning approaches. (1) Location-based classification: Models are trained and tested on data from specific anatomical regions (e.g., distal vein, Anastomosis), allowing for targeted evaluation of localized flow characteristics. (2) Universal classification: Data from all anatomical locations are combined, with location metadata included as an additional feature, enabling models to generalize across heterogeneous vascular environments. (3) Patient-level classification: Predictions from multiple recordings for the same patient are aggregated to generate a single diagnostic outcome, reflecting a clinically realistic scenario. Applying these three scenarios to both classical and deep learning models enables a direct apple to apple comparison of their discrimination performance, generalizability, and potential clinical utility.

## 3. Results

**Figure 3:**
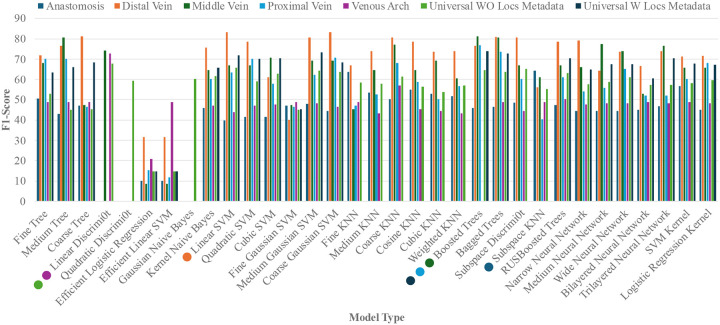
F1-score performance of 33 classical machine learning models for AVF stenosis classification across five anatomical locations and two universal scenarios (with and without location metadata). Colored circle markers indicate the best-performing model for each scenario: Anastomosis (blue), Distal Vein (orange), Middle Vein (dark green), Proximal Vein (light blue), Venous Arch (purple), Universal without location metadata (light green), and Universal with location metadata (dark blue).

To compare classical machine learning and deep learning approaches, we focus on the best-performing model for each scenario. For classical models, the top model at each anatomical location was selected based on the highest F1 score during test evaluation. For the deep learning approach, ViT model trained on 374×128 two-dimensional mel-spectrograms, as reported in [32], is used as the reference best-performing model. All classical machine learning models were systematically evaluated across five anatomical locations and universal scenarios (with and without location metadata), resulting in a total of 231 model evaluations. For location-based classification, each model’s F1 score was calculated at the Distal Vein, Middle Vein, Proximal Vein, Anastomosis, and Venous Arch sites. For the universal models, F1 scores were computed for models trained on all anatomical locations, both with and without location metadata. Figure 3 presents the F1 scores for all 33 classical learning models across these different scenarios, highlighting the variability in performance across locations, the impact of including anatomical metadata, and the relative performance of different algorithmic families.

### 3.1 Model Training

To address class imbalance in the classical machine learning models, we applied a misclassification cost, which penalizes errors in the minority class more heavily. This approach, through internal adjustment of class priors and observation weights, effectively mimics the impact of oversampling the minority class, as implemented in the original study[32]. Classical models were trained using MATLAB’s Classification Learner App, which follows a standard supervised learning workflow: input features were split into 70% training, 15% validation, and 15% test hold-out sets, and 10-fold cross-validation was applied to optimize model hyperparameters and evaluate performance. The optimizer and learning method were automatically selected by the app based on the algorithm type, as detailed in SI.2. For the deep learning approach, the Vision Transformer (ViT) model was trained using the Adam optimizer with a weighted binary cross-entropy loss function, and 10-fold cross-validation was applied to monitor performance and select the best-performing model. Weighing the loss function accounted for class imbalance by emphasizing the minority class during optimization, ensuring that both stenotic and patent samples contributed appropriately to model updates.

### 3.2 Performance Metrics

Given the strong class imbalance in the dataset, overall accuracy was not considered a reliable measure of model performance. Following the reporting standards of the original deep learning study [32], performance metrics were computed as follows:

- **Location-based models:** Precision, Recall, and F1 Score were calculated for the stenotic (positive) class, in addition to the area under the receiver operating characteristic curve (AUROC) and the area under the precision-recall curve (AUPRC).
- **Universal models:** Only AUROC and AUPRC were reported, consistent with the original study, as threshold-dependent metrics were not evaluated for models trained on combined anatomical data.

For the ViT model in location-based experiments, precision was not originally reported. To provide a complete comparison, precision was calculated from the reported Recall and F1 Score values using the harmonic mean relationship:

(1) $$\text{Precision}=\frac{F1\times \text{Recall}}{2\times \text{Recall}-F1}$$

This set of metrics allows for a consistent and interpretable comparison of classical machine learning models and the ViT across different anatomical locations and universal scenarios, while accounting for class imbalance.

### 3.3 Observation-Based Performance

#### 3.3.1 Location-Based Model Performance

We first examined model performance across the five anatomical AVF locations to assess the ability of classical machine learning and ViT models to discriminate between patent and stenotic samples under varying class prevalence. As reported in Tables 1 and 2, overall, classical machine learning consistently matches or outperforms the ViT model in key discrimination metrics while achieving substantially higher precision across all sites.

- **Distal Vein** (46% stenotic, near balanced): The Linear SVM achieved superior overall discrimination with AUROC of 0.877 and AUPRC of 0.879, exceeding the ViT (AUROC 0.76, AUPRC 0.72). This model also attained higher precision (85.95% vs. 83.0%). ViT achieves a higher F1 score (89.1% vs. 83.14%) due to its elevated recall (96.1% vs. 82.82%), but this comes at the cost of increased false positives, highlighting the trade-off between recall and precision.
- **Middle Vein** (11.1% stenotic): The class imbalance poses a challenge, yet classical ML using Boosted Trees demonstrates robust performance, with AUROC 0.939, AUPRC 0.915, and a well-balanced F1 of 81.33% (precision 78.65%, recall 84.87%). In contrast, the ViT exhibits extremely low F1 (40.0%) due to poor precision (25.0%), despite perfect recall (100%), indicating a tendency to over-classify stenotic cases in this highly imbalanced location.
- **Proximal Vein** (15.7% stenotic): Classical ML (Ensemble Boosted Trees) again shows superior performance, achieving balanced precision and recall (76.87% each, F1 76.87%) and strong AUROC/AUPRC values (0.936/0.937). ViT demonstrates higher recall (84.6%) but low precision (44.0%), resulting in a reduced F1 of 57.9%, consistent with the tendency of deep learning to over-predict the minority class under moderate imbalance.
- **Anastomosis** (11.5% stenotic): Classical ML (Ensemble Subspace KNN) attains the highest precision across all locations (95.45%) alongside robust AUROC (0.949) and AUPRC (0.939), markedly surpassing the ViT (precision 72.9%, AUROC 0.64, AUPRC 0.14). This demonstrates the advantage of feature-based classical approaches in locations with moderate class imbalance.
- **Venous Arch** (3.2% stenotic): Even in extreme imbalance, classical Linear Discriminant maintains strong discrimination (AUROC 0.873, AUPRC 0.793), achieving high recall (95.45%) and F1 (72.62%) while outperforming ViT in precision (51.8% vs. 72.62%). ViT’s low precision in this location highlights its susceptibility to false positives in rare-class settings.

#### 3.3.2 Universal Models

As reported in Table 3, Clasical ML models also outperform ViT in both universal configurations (With and Without Location Metadata). The improvement is especially pronounced when location metadata is incorporated (AUROC 0.893 vs. 0.82; AUPRC 0.890 vs. 0.54), highlighting the importance of anatomical context. While both approaches benefit from metadata inclusion, the performance gain is more substantial for ensemble-based classical models such as Boosted Trees.

#### 3.3.3 Overall Insights

**Figure 4:**
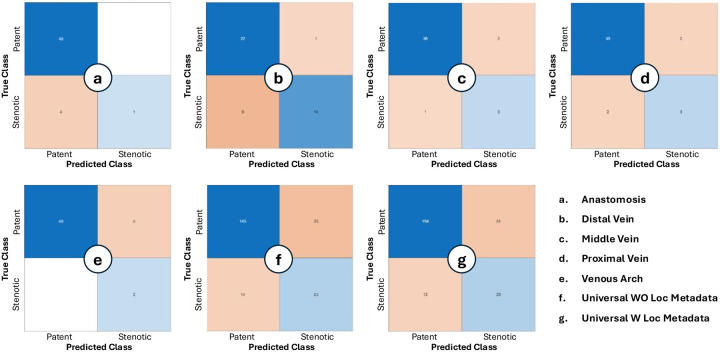
Confusion matrices of the best classical ML models for AVF stenosis classification across the five anatomical locations—(a) Anastomosis, (b) Distal Vein, (c) Middle Vein, (d) Proximal Vein, and (e) Venous Arch—as well as for the universal models (without (f) and with (g) metadata).

**Figure 5:**
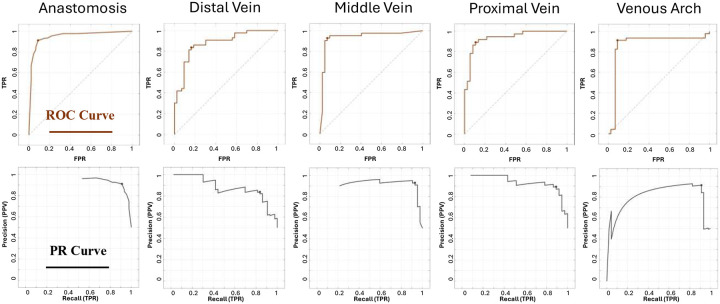
ROC and Precision-Recall (PR) curves of the best classical ML models for AVF stenosis classification across the five anatomical locations.

Across all five anatomical locations and both universal configurations (with and without location metadata), classical machine learning models consistently outperform the Vision Transformer (ViT) model, delivering more balanced and clinically meaningful predictions with higher precision and substantially better AUROC and AUPRC scores. The advantage of classical ML is particularly evident in the universal setting when anatomical location metadata is incorporated (AUROC 0.893 vs. 0.82; AUPRC 0.890 vs. 0.54). While both approaches benefit from metadata inclusion, ensemble-based classical models such as Boosted Trees show the most significant performance gains. These results demonstrate that classical models, when combined with carefully selected acoustic features, offer superior reliability, precision, and interpretability for AVF stenosis detection. Figure 4(a–e) shows the confusion matrices of the best classical ML models across the five anatomical locations, as well as both universal models (with and without metadata). The corresponding ROC and precision-recall curves for the location-specific and universal models are presented in Figure 5.

### 3.4 Patient Level Classification

At the patient level, a patient is classified as “stenotic” if any stenotic lesion is present anywhere along their arteriovenous fistula. Conversely, a patient is considered “patent” only if no stenotic lesions are detected at any location[32]. For the overall predicted label, each individual location-based model must predict patent at every site for the patient to be classified as patent; if any location-based model predicts stenosis, the patient is classified as stenotic. For each location, the best-performing model reported in Figure 3 is used to generate predictions. Table 4 provides an “apple-to-apple” comparison between the classical machine learning models and the ViT model reported in[32]. As before, precision for the ViT model is calculated from the reported F1 Score and Recall values. Both models demonstrate very similar overall performance, with identical F1 scores (0.91) and specificity (0.79). The main difference lies in a slight trade-off between precision and recall: the traditional machine learning model achieves 2% higher precision (0.91 vs. 0.89), indicating very slightly fewer false positives, while the ViT model shows 1% higher recall (0.92 vs. 0.91), suggesting very slightly improved sensitivity in detecting positive cases. Overall, these differences are negligible, and the two approaches can be considered practically equivalent for patient level classification of AVF.

### 3.5 Interpretability and Explainability Analysis

To interpret the decision-making mechanisms of the classical machine learning models and relate their predictions to physically and clinically meaningful acoustic phenomena, we employed both global and local explainability methods based on SHAP (SHapley Additive exPlanations)[44] and LIME [36]. SHAP is grounded in cooperative game theory[47], where the Shapley value assigns a contribution to each feature by considering its marginal effect across all possible feature subsets. Formally, the Shapley value for feature i is defined as:

(2) $$\varphi_{i}=\sum_{S\subseteq F\backslash \{i\}}\frac{\left|S\right|!\left(\left|F\right|-\left|S\right|-1\right)!}{\left|F\right|!}[f\left(S\cup \left\{i\right\}\right)-f(S)]$$

where *F* is the full feature set and *S* represents a subset of features. This formulation ensures a fair and consistent attribution of feature contributions to the model output.

Within this framework, global SHAP importance is computed as the mean absolute Shapley value across all samples, providing a ranking of features based on their overall influence (visualized as SHAP importance bar plots). SHAP summary plots further extend this by showing the distribution of feature effects across all samples, capturing both the magnitude and direction of influence. In contrast, local SHAP explanations quantify how individual feature values contribute to a single prediction, enabling case-specific interpretation. Complementarily, LIME (Local Interpretable Model-agnostic Explanations) approximates the model locally with an interpretable surrogate model to explain individual predictions, offering an alternative perspective on local behavior.

These analyses are reported for the best-performing location-based model (distal vein) and the best-performing universal model (with location metadata), as these models demonstrated the highest overall performance and are representative of the two key classification scenarios investigated in this study. This selection enables a focused yet comprehensive evaluation of model behavior across both localized and generalized settings, with detailed results of SHAP importance, SHAP summary plots, and local explanations presented in the following section.

#### 3.5.1 Global Feature Importance and Physiological Interpretation for Distal Vein model

**Figure 6:**
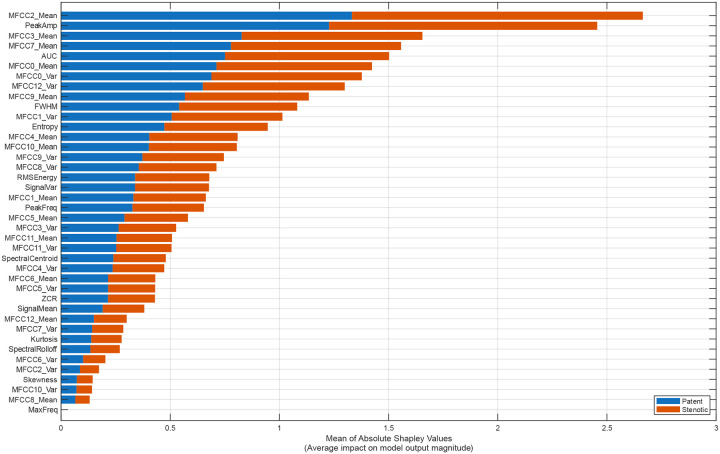
Global feature importance ranking for the best classical machine learning model at the distal vein location, based on mean absolute SHAP (Shapley) values. The plot highlights the dominant influence of low-order MFCC coefficients (especially MFCC2 Mean and MFCC3 Mean), Peak Amplitude, and AUC, reflecting key spectral envelope and acoustic energy features associated with stenotic blood flow.

Figure 6 presents the global feature importance of ranking for the distal vein model based on the mean absolute Shapley value of each predictor. This metric reflects the average magnitude of a feature’s influence on the model output, independent of prediction direction. The results demonstrate a strongly hierarchical structure, with a small subset of acoustic features dominating the decision process.

The most influential predictor is MFCC2 Mean, followed by Peak Amplitude (PeakAmp) and MFCC3 Mean, all of which show substantially higher impact than the remaining features. These are followed by a second tier including MFCC7 Mean, area under the curve (AUC), and MFCC0 Mean, while higher-order frequency descriptors such as MaxFreq, Skewness, and Spectral Rolloff exhibit minimal influence.

From a physiological perspective, the dominance of low-order MFCC coefficients is particularly meaningful. MFCCs encode the spectral envelope of the signal, reflecting how acoustic energy is distributed across frequency bands. In vascular acoustics, this envelope is influenced by blood flow velocity, vessel geometry, and turbulence intensity. Lower-order MFCCs are especially sensitive to global spectral shifts that occur when laminar flow transitions to disturbed or turbulent regimes in stenotic segments.

PeakAmp and AUC, representing instantaneous and integrated acoustic energy, further support this interpretation. Elevated acoustic energy is a hallmark of stenosis, resulting from increased flow velocity and vortex formation in narrowed vessels. The relatively low importance of MaxFreq and Spectral Rolloff suggests that overall spectral structure and energy distribution, rather than extreme high-frequency components, are the primary discriminative cues in the distal vein.

#### 3.5.2 Directional Effects and Acoustic Signatures of Stenosis of Distal Vein Model

**Figure 7:**
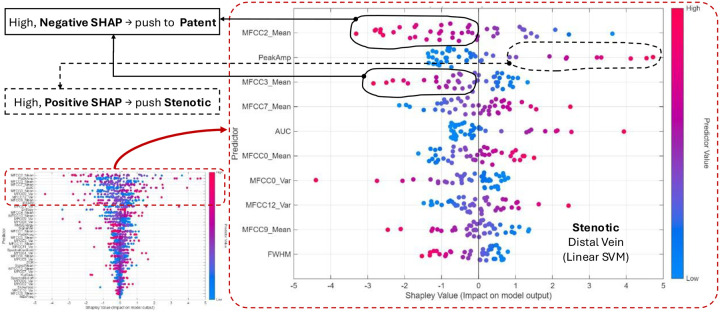
SHAP summary plot for the best classical machine learning model at the distal vein location, showing the impact of individual acoustic features on model predictions for patent versus stenotic classes. Higher values of MFCC2 Mean and MFCC3 Mean drive predictions toward the patent class, while higher Peak Amplitude and AUC strongly contribute to stenotic predictions.

Figure 7 (Large version in **Figure SI.3.1**) shows the SHAP summary plots for the distal vein model, illustrating how individual feature values influence predictions toward either the patent or stenotic class. For MFCC2 Mean and MFCC3 Mean, higher values are consistently associated with negative SHAP values, pushing predictions toward the patent class, while lower values strongly promote stenotic predictions. This inverse relationship suggests that stenosis is associated with a flattening or downward shift of the spectral envelope, consistent with broadband turbulence and loss of harmonic structure. In contrast, PeakAmp and AUC exhibit the opposite behavior: higher values contribute positively toward stenotic classification. This aligns with the expected increase in acoustic energy caused by turbulent jet formation in stenotic regions. FWHM indicates that broader frequency bandwidths favor patent predictions, suggesting that healthy flow produces more evenly distributed spectral content, whereas stenotic flow concentrates energy into narrower bands. The relatively limited importance of PeakFreq further indicates that stenosis detection relies more on global spectral patterns than on a single dominant frequency. The broader SHAP value distribution for MFCC2 Mean compared to low-impact features confirms that a small subset of acoustically meaningful descriptors drives most model decisions.

#### 3.5.3 Local SHAP Explanations and Error Analysis of Distal Vein Model

**Figure 8:**
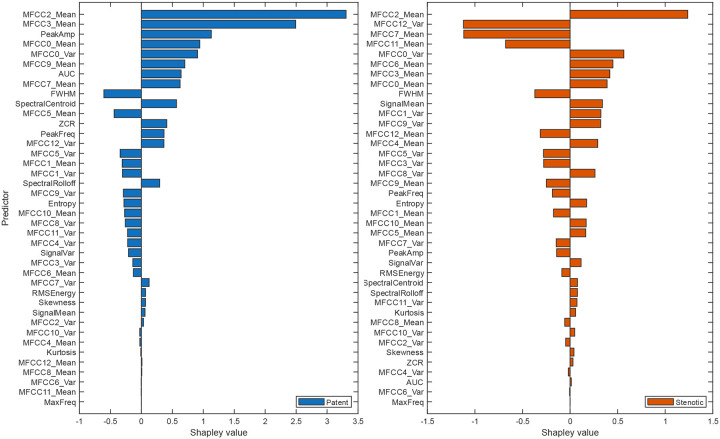
Local SHAP explanations for distal vein cases. (Left) Correctly classified patent sample, where spectral features (MFCC2, MFCC3) reflect stable, laminar flow. (Right) False-positive case, where a patent sample is misclassified as stenotic because spectral irregularities (MFCC2 Mean, MFCC0 Var) acoustically mimic the signatures of hemodynamic turbulence.

Figure 8 **(left)** provides a local SHAP explanation for a correctly classified patent sample from the Distal Vein model. The prediction is supported by a coherent pattern where major features, such as MFCC2 Mean and MFCC3 Mean, exert strong contributions toward the patent class. From a physiological perspective, these low-order coefficients indicate a spectral envelope consistent with stable, laminar flow. The model’s confidence is further reinforced by low values for Peak Amp and AUC, reflecting the reduced acoustic energy and turbulence characteristic of an unobstructed vessel.

In contrast, Figure 8 **(right)** illustrates a false-positive case, where a patent sample was incorrectly classified as stenotic. In this instance, features like MFCC2 Mean and MFCC0 Var exert strong positive SHAP contributions toward the stenotic class. This error occurs because the signal exhibits elevated high-frequency content without a corresponding increase in energy, which the model interprets as a sign of vascular narrowing. Such misleading acoustic patterns often arise from benign flow irregularities, anatomical variability, or sensor positioning effects that acoustically mimic the signatures of hemodynamic turbulence. This analysis highlights a specific limitation where physical disturbances unrelated to stenosis can trigger false positives, underscoring the importance of combining high precision with interpretable insights to distinguish true lesions from acoustic mimics.

#### 3.5.4 Limitations of LIME for Vascular Acoustic Signals

**Figure 9:**
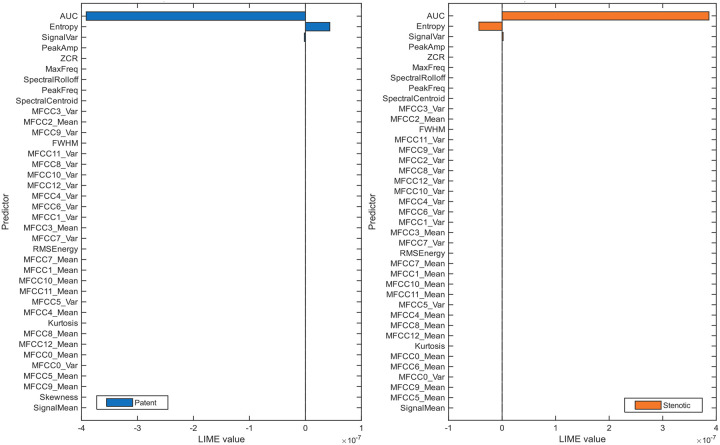
LIME explanations for two cases from the distal vein model. (Left) Correctly classified patent sample. (Right) Misclassified stenotic sample. In both instances, LIME highlights only one or two dominant features while assigning near-zero importance to the rest, in contrast to the more distributed and physiologically consistent attributions provided by SHAP.

Figure 9 **(left and right)** presents LIME explanations for a correctly classified patent sample and a misclassified stenotic sample, respectively. In both cases, LIME assigns importance to only one or two features while ignoring the rest. This sparse attribution contrasts with SHAP results, which consistently demonstrate that multiple features jointly contribute to predictions. The limitation arises from LIME’s reliance on local linear approximations, which fail to capture the complex, nonlinear interactions inherent in vascular acoustic signals. Consequently, LIME provides less reliable explanations in this context, whereas SHAP offers a more faithful and physiologically consistent interpretation.

#### 3.5.5 The Role of Anatomical Context in Universal Model

**Figure 10:**
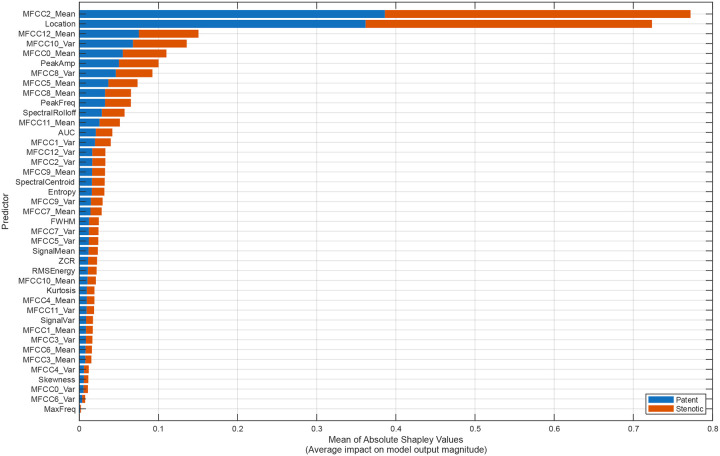
Global feature importance for the universal model with location metadata, based on mean absolute SHAP values. MFCC2 Mean and Location are the two most influential predictors. Importance drops sharply thereafter, with only MFCC12 Mean, MFCC10 Var, MFCC0 Mean, MFCC8 Var, and PeakAmp showing moderate contribution.

Figure 10 Global feature importance ranking for the universal classical machine learning model (with anatomical location metadata), based on mean absolute SHAP values. MFCC2 Mean and Location are the two most influential predictors. After these two dominant features, the importance drops sharply, with MFCC12 Mean, MFCC10 Var, MFCC0 Mean, MFCC8 Var, and PeakAmp forming a second tier of moderate importance, while the remaining features contribute far less.

This hierarchical pattern indicates that the universal model relies primarily on anatomical context and the low-order spectral envelope captured by MFCC2 Mean. Physiologically, the strong contribution of Location reflects fundamental differences in baseline hemodynamics, vessel diameter, flow velocity, and wall properties across AVF segments. MFCC2 Mean, which encodes coarse spectral shape, remains highly important because it effectively captures global shifts in acoustic energy distribution associated with the transition from laminar to turbulent flow in stenotic regions. The rapid decrease in feature importance beyond these top predictors suggests that a small subset of physically meaningful descriptors — primarily related to spectral envelope and overall energy — drives most of the model’s decisions, while higher-order or more granular features add only marginal value in the universal setting.

**Figure 11:**
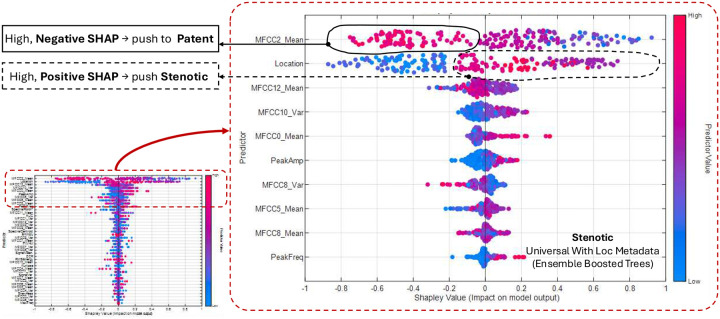
SHAP summary plot for the stenotic class. Higher MFCC2 Mean values push predictions toward the Patent class, reflecting the stable spectral envelope of laminar flow. Conversely, higher values of Location, PeakAmp, and MFCC-related variability drive the model toward a Stenosis classification. These trends capture the concentration of lesions at high-stress anatomical sites and the transition to turbulent flow, characterized by high-energy bruits and chaotic vortex shedding.

Figure 11 (Large version in **Figure SI.3.2**) illustrates the SHAP distributions for the stenotic class, revealing how higher feature values drive the model’s classification through specific hemodynamic mechanisms. A key finding is that higher MFCC2 Mean values exert a strong negative SHAP impact, pushing the prediction toward the Patent class. Physiologically, higher MFCC2 values represent a stable and broad spectral envelope characteristic of the smooth, laminar blood flow found within a healthy, unobstructed vessel. In contrast, higher values for the Location feature—representing specific anatomical segments like the juxta-anastomotic area—act as positive predictors for stenosis. This aligns with clinical reality, as these high-stress vascular sites are most susceptible to intimal hyperplasia and subsequent narrowing.

Similarly, higher PeakAmp values promote a stenosis prediction by capturing the physical manifestation of a vascular bruit. As blood is forced through a narrowed lumen, its velocity increases significantly, generating high-energy acoustic signatures that are absent in patent vessels. This is further supported by higher MFCC-related variability, which indicates a non-stationary and chaotic signal. Physically, this variability corresponds to the transition from steady flow to turbulent vortex shedding; while a patent fistula produces a rhythmic, consistent sound, the unstable acoustic nature of a stenotic lesion results in high spectral variance. Higher Total Energy levels also contribute to positive predictions, likely reflecting the high-pressure flow dynamics required to maintain circulation across a significant blockage. Ultimately, the universal model reveals a coherent decision structure where high-value features act as direct proxies for the physical disturbances caused by vascular narrowing, mirroring the diagnostic logic used by clinicians during physical examinations.

## 4. Discussion

The findings of this study highlight the continued value of classical ML approaches in specialized medical diagnostics, especially when compared to the increasingly opaque “black-box” nature of deep learning models. While the Vision Transformer represents a state-of-the-art solution in computer vision, our analysis shows that classical ML models, when combined with carefully engineered acoustic features, often perform as well as—or even better than—DL methods in detecting arteriovenous fistula (AVF) stenosis. This is particularly evident in site-specific detection: for instance, at the Anastomosis and Venous Arch regions, classical ensemble models achieved markedly higher precision than the ViT, which tended to over-predict stenotic cases. In a clinical context, this distinction is critical. Models with high precision can reduce false positives and facilitate earlier detection of AVF stenosis, thereby supporting timely intervention and optimizing the use of confirmatory diagnostic procedures such as duplex ultrasound and angiography. Similarly, models with high recall reduce false negatives to ensure patients are properly treated when stenosis occurs.

Beyond performance metrics, classical models offer a major advantage in transparency and interpretability. Using Explainable AI (XAI) tools such as SHAP, we were able to uncover which acoustic features—like Peak Amplitude and low-order Mel-frequency cepstral coefficients (MFCCs)—most strongly influenced the model’s predictions of stenosis. This insight transforms the model from a black box into a clinically informative tool. When high-energy turbulence or specific spectral changes are flagged as the basis for a positive prediction, it aligns with known physiological patterns of hemodynamic disruption caused by vessel narrowing. Such alignment fosters trust in medical AI, as clinicians are more likely to rely on recommendations when the reasoning behind them resonates with established diagnostic principles.

At the patient level, the results are equally encouraging. When evaluating overall vascular access health, both classical and deep learning approaches reached comparable F1 scores of 0.91. However, the classical models’ ability to maintain high precision and recall while achieving this accuracy makes them especially suitable for point-of-care screening. By incorporating metadata such as the anatomical location of recordings, these models offer a “universal” diagnostic approach that accounts for natural hemodynamic differences between regions like the distal vein and venous arch. Overall, our study suggests that for critical applications such as AVF monitoring, clinical adoption may benefit more from interpretable, reliable models grounded in physiological reality than from increasingly complex architectures.

## 5. Conclusion

This study demonstrates that, although deep learning models such as the Vision Transformer excel in automated feature extraction, classical machine learning models remain highly effective—and in many cases superior—for the task of acoustic AVF stenosis detection. By incorporating expert-engineered features and anatomical metadata, classical models achieved a patient-level F1 score of 0.91 and an AUROC of 0.893, matching the performance of more complex architectures like ViT, while maintaining higher precision at specific anatomical sites. This combination of high accuracy and low false-positive rates is essential for reducing unnecessary clinical interventions and optimizing healthcare resource utilization.

The use of Explainable AI through SHAP analysis further enhances the value of the classical approach by providing a transparent connection between acoustic features and vascular physiology. Key predictors, including Peak Amplitude and selected MFCC coefficients, align with known hemodynamic patterns, fostering clinician trust in model outputs. These findings indicate that, for point-of-care vascular monitoring, clinical adoption is best supported not by increasing model complexity, but by developing interpretable, reliable, and high-performing systems that prioritize both physician trust and patient safety. Such an approach establishes a strong foundation for the future of non-invasive, AI-assisted surveillance of vascular access.

## Figures and Tables

**Table 1:** Performance of the best deep learning model (ViT) for AVF stenosis classification at each anatomical location. The ViT model with 374×128 mel-spectrogram input was the best-performing deep learning approach in the original study at all anatomical location.

Location	Best Model	Precision *	Recall	F1 Score	AUROC
*Distal Vein*	ViT (374×128)	83.0%	96.1%	89.1%	0.76
*Middle Vein*	ViT (374×128)	25.0%	100.0%	40.0%	0.85
*Proximal Vein*	ViT (374×128)	44.0%	84.6%	57.9%	0.82
*Anastomosis*	ViT (374×128)	72.9%	94.1%	82.1%	0.64
*Venous Arch*	ViT (374×128)	51.8%	93.8%	66.7%	0.76

* Precision is not reported in Reference paper, so the current pensions are calculated by Recall and F1 score based on Eq (1).

**Table 2:** Performance of the selected best classical machine learning models for AVF stenosis classification at each anatomical location.

Location	Best Model	Precision	Recall	F1 Score	AUROC	AUPRC
*Distal Vein*	Linear SVM	85.95%	82.82%	83.14%	0.87723	0.87923
*Middle Vein*	Ensemble Boosted Trees	78.65%	84.87%	81.33%	0.93878	0.91487
*Proximal Vein*	Ensemble Boosted Trees	76.87%	76.87%	76.87%	0.93572	0.93675
*Anastomosis*	Ensemble Subspace KNN	95.45%	60%	64.29%	0.94864	0.93866
*Venous Arch*	Linear Discriminant	66.66%	95.45%	72.62%	0.87335	0.79317

**Table 3:** Performance comparison of the best classical machine learning models and the Vision Transformer (ViT) model in universal configurations for AVF stenosis classification, with and without location metadata.

Location	Best Model	Precision	Recall	F1 Score	AUROC	AUPRC
Universal (With Loc Metadata)	*Classic:* Ensemble Boosted Trees	71.9%	77%	73.83%	0.89309	0.89023
Universal (Without Loc Metadata)	*Classic:* Linear Discriminant	66.14%	71.81%	67.75%	0.7785	0.70629
Universal (With Loc Metadata)	*Deep:* ViT with 374×128 input	NR*	NR*	NR*	0.82	0.54
Universal (Without Loc Metadata)	*Deep:* ViT with 374×128 input	NR*	NR*	NR*	0.68	0.28

* NR = Not Reported in Reference paper.

**Table 4:** Patient-level performance comparison between the best classical machine learning models and the Vision Transformer (ViT) model for AVF stenosis classification.

Model	Precision	Recall	Specificity	F1-D
**Classical ML**	0.91	0.91	0.79	0.91
**ViT Model**	0.89	0.92	0.79	0.91

## Data Availability

The trained models produced by this study will be made available by the authors upon reasonable request.
